# Bioactive Extracts from Cyclodextrin-Elicited *Morus alba* Roots Reduce MCF-7 Breast Cancer Cell Viability

**DOI:** 10.3390/plants15213255

**Published:** 2026-10-22

**Authors:** Francisco José Vidal-Sánchez, Irene Conesa, Miriam Romero-Muñoz, Silvia Navarro-Orcajada, Carmen López-Sierra, José Manuel López-Nicolás, Margarita Pérez-Jiménez

**Affiliations:** 1 Department of Biochemistry and Molecular Biology-A, Faculty of Biology, University of Murcia, Regional Campus of International Excellence “Campus Mare Nostrum”, 30100 Murcia, Spain; fjose.vidal@um.es (F.J.V.-S.); irene.conesav@um.es (I.C.); josemln@um.es (J.M.L.-N.); 2 Department of Plant Biotechnology, Genomics and Breeding, Instituto Murciano de Investigación y Desarrollo Agrario y Alimentario (IMIDA), 30150 Murcia, Spain; miriam.romero3@carm.es (M.R.-M.); c.lopezsierra@um.es (C.L.-S.)

**Keywords:** antioxidant activity, elicitation, *Morus alba*, stilbenes, oxyresveratrol, moracin M, MCF-7, cell viability

## Abstract

Plant in vitro cultures offer controlled systems for producing bioactive phenolics, yet the effects of elicitation on extract bioactivity remain insufficiently characterised. This study evaluated hydroxypropyl-β-cyclodextrin (HP-β-CD; 0–50 mM) elicitation in *Morus alba* cv. Filipina adventitious roots, examining biomass, extracellular oxyresveratrol and moracin M accumulation, antioxidant capacity and effects on MCF-7 cell viability. Elicitation reduced final root dry weight by 82–93% relative to non-elicited controls. After 21 days, 25 mM HP-β-CD yielded the highest oxyresveratrol and moracin M concentrations, reaching 61.79 and 31.78 μM, respectively, approximately 73- and 61-fold the control values. This treatment also produced the highest total phenolic content and ABTS antioxidant capacity, with the latter reaching 183.44 μM Trolox equivalents compared with 59.26 μM in the control. Extracts from elicited cultures produced a non-monotonic MTT response. At 8% (*v*/*v*), the CD25-derived extract reduced MCF-7 viability to 22.6% of the vehicle control after 48 h, accompanied by reduced cell attachment and altered morphology. These findings support HP-β-CD elicitation as a strategy for obtaining phenolic-rich extracellular extracts with antioxidant activity and measurable effects on MCF-7 cell viability.

## 1. Introduction

Plants are a major source of structurally diverse bioactive compounds with recognised roles in human nutrition and health, among which phenolic compounds have attracted particular attention due to their biological properties [1,2]. However, the production of these metabolites from field-grown material is inherently variable and poorly controllable, limiting both yield and reproducibility. Plant in vitro culture systems have therefore emerged as alternative platforms that enable the controlled production of specialised metabolites under defined conditions [3].

Adventitious root cultures provide a robust and scalable in vitro system, combining genetic and biochemical stability with high metabolic capacity [4]. Their compatibility with liquid culture not only facilitates scale-up but also provides direct access to extracellular metabolites, enabling elicitation-driven changes to be investigated at the extract level. Accordingly, elicitation strategies have been widely applied to redirect metabolic fluxes and promote both the accumulation and release of phenolic compounds in response to controlled stimuli [5].

Cyclodextrins (CDs) have emerged as effective elicitors in plant in vitro systems, combining the stimulation of secondary metabolism with the ability to form inclusion complexes with hydrophobic metabolites [6]. In particular, β-CD derivatives such as 2-hydroxypropyl-β-cyclodextrin (HP-β-CD) are widely used due to their high aqueous solubility and strong interaction with phenolic compounds [7]. Beyond elicitation, CDs act as molecular carriers that enhance solubility, stabilisation and, importantly, the extracellular accumulation of phenolic metabolites, thereby influencing the composition of culture supernatants under elicitation conditions [8]. HP-β-CD was selected because its ability to promote extracellular phenolic accumulation has been demonstrated in plant culture systems [9]. Previous work in mulberry has also established the potential of CD-based elicitation, including combinations with other elicitors [10,11]. Evaluating HP-β-CD alone allowed its concentration-dependent effects to be examined without interactions with additional elicitors, providing a defined basis for linking extracellular metabolite accumulation with extract bioactivity.

*Morus alba* L. is a well-recognised source of phenolic compounds with reported biological activities. Stilbenes such as oxyresveratrol and benzofuran derivatives, including moracin M, are among its major bioactive constituents, associated with antioxidant and antiproliferative properties [12]. Notably, moracin-type compounds have been proposed to originate from stilbenoid precursors, suggesting a biosynthetic and functional linkage between these phenolic classes [13]. While extensively studied as isolated molecules, *M. alba* in vitro systems offer a suitable model to investigate how elicitation modulates phenolic production and release at the extract level [10].

Despite the widespread use of plant in vitro systems to enhance metabolite production, most studies remain focused on biomass-associated accumulation or on individual compounds in isolation. In parallel, bioactivity assessments are typically performed using purified metabolites or plant extracts that are not directly linked to controlled production systems, resulting in a disconnect between metabolite production and functional evaluation. Consequently, the biological relevance of elicitation-driven metabolic changes is often assumed rather than experimentally demonstrated. In particular, the extracellular metabolite pool generated under elicitation conditions, despite its direct accessibility and potential functional relevance, remains underexplored. Furthermore, the temporal dynamics of metabolite accumulation are rarely considered, limiting the identification of optimal elicitation windows and hindering the interpretation of how production profiles relate to biological activity.

To determine whether these elicitation-driven changes translate into functional biological effects, appropriate cell-based models are required. MCF-7 cells represent a well-established oestrogen receptor-positive breast cancer model widely used for the preliminary assessment of changes in cell viability induced by natural compounds and complex botanical extracts [14]. Several *M. alba* phenolics have been associated with antiproliferative effects [15]; however, the activity of extracellular extracts generated through controlled elicitation remains poorly characterised. Evaluating these extracts in MCF-7 cells, therefore, provides a functional readout of whether enhanced phenolic production is accompanied by measurable effects in a tumour cell model.

This study aimed to evaluate the effect of CD-mediated elicitation on secondary metabolism in *M. alba* adventitious root cultures and to determine whether elicitation-driven changes in metabolite production were accompanied by measurable functional effects. Adventitious roots were elicited with increasing concentrations of HP-β-CD, and biomass accumulation, extracellular production of oxyresveratrol and moracin M, and total phenolic content were analysed over time. The antioxidant capacity of the resulting culture supernatant extracts was also determined, and their effects on MCF-7 breast cancer cell viability were evaluated as a functional readout of extract bioactivity.

## 2. Results

### 2.1. Biomass Accumulation of Morus alba Adventitious Root Cultures

**Figure 1:**
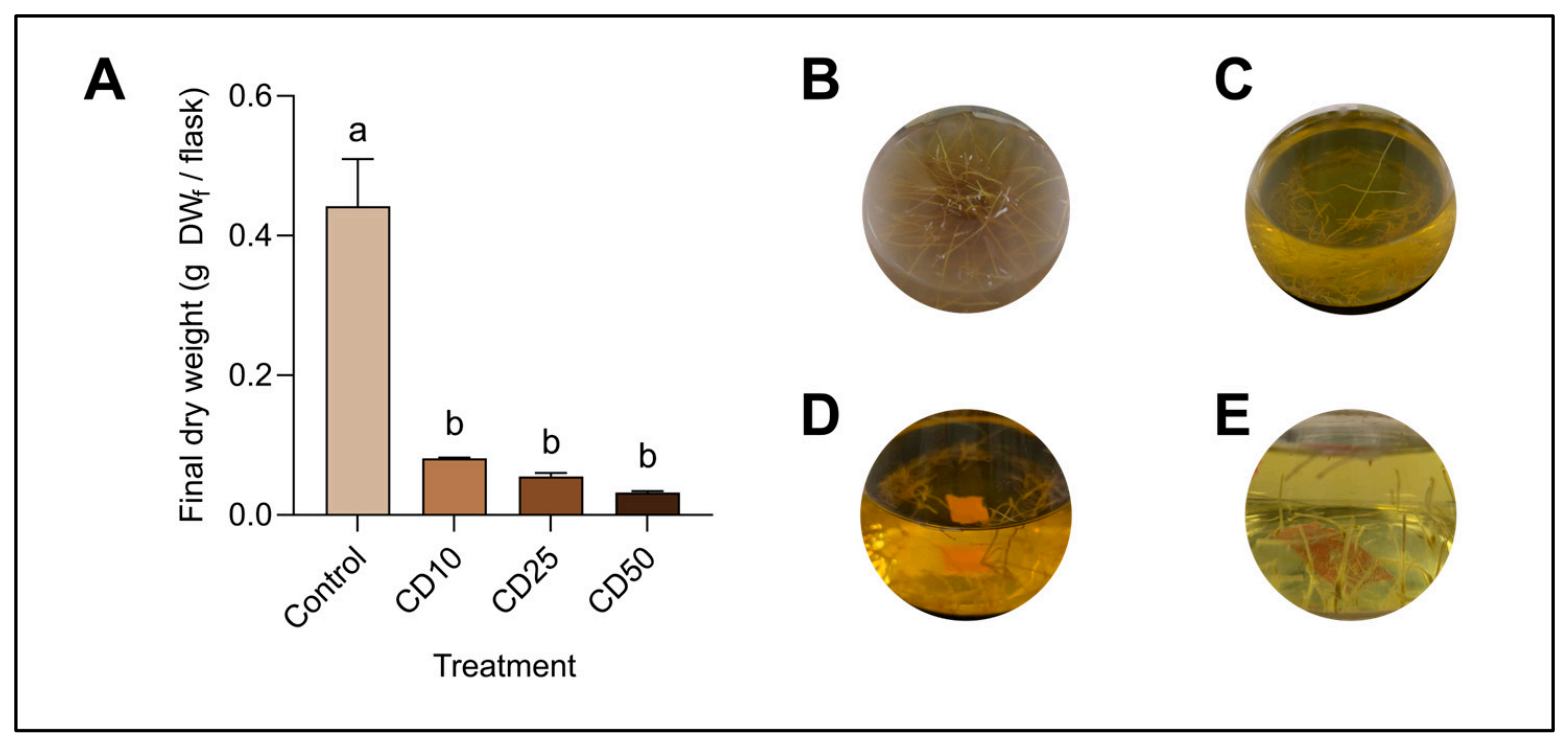
Effect of HP-β-CD elicitation on biomass accumulation and culture appearance of *M. alba* adventitious roots after 21 days of elicitation. (**A**) Final root dry weight (DW_f_), expressed as mean ± SEM (*n* = 4). Different letters indicate statistically significant differences among treatments (one-way ANOVA followed by Tukey’s multiple comparisons test, *p* < 0.05). (**B**–**E**) Representative photographs of cultures treated with 0, 10, 25 and 50 mM HP-β-CD, respectively.

Final root dry weight (DW_f_) is presented in Figure 1A. Control cultures reached the highest DW_f_ (0.44 g per flask). In contrast, all CD-elicited cultures had significantly lower DW_f_ than the control, with reductions ranging from 82% to 93%. No significant differences in DW_f_ were detected among the three elicited treatments. Photographs taken after 21 days of elicitation illustrate differences in culture appearance (Figure 1B–E). The culture medium appeared brownish in the control, yellow at 10 and 50 mM HP-β-CD and intensely orange at 25 mM.

### 2.2. Time-Course and Kinetic Modelling of Phenolic Compound Accumulation

#### 2.2.1. Oxyresveratrol Accumulation

**Figure 2:**
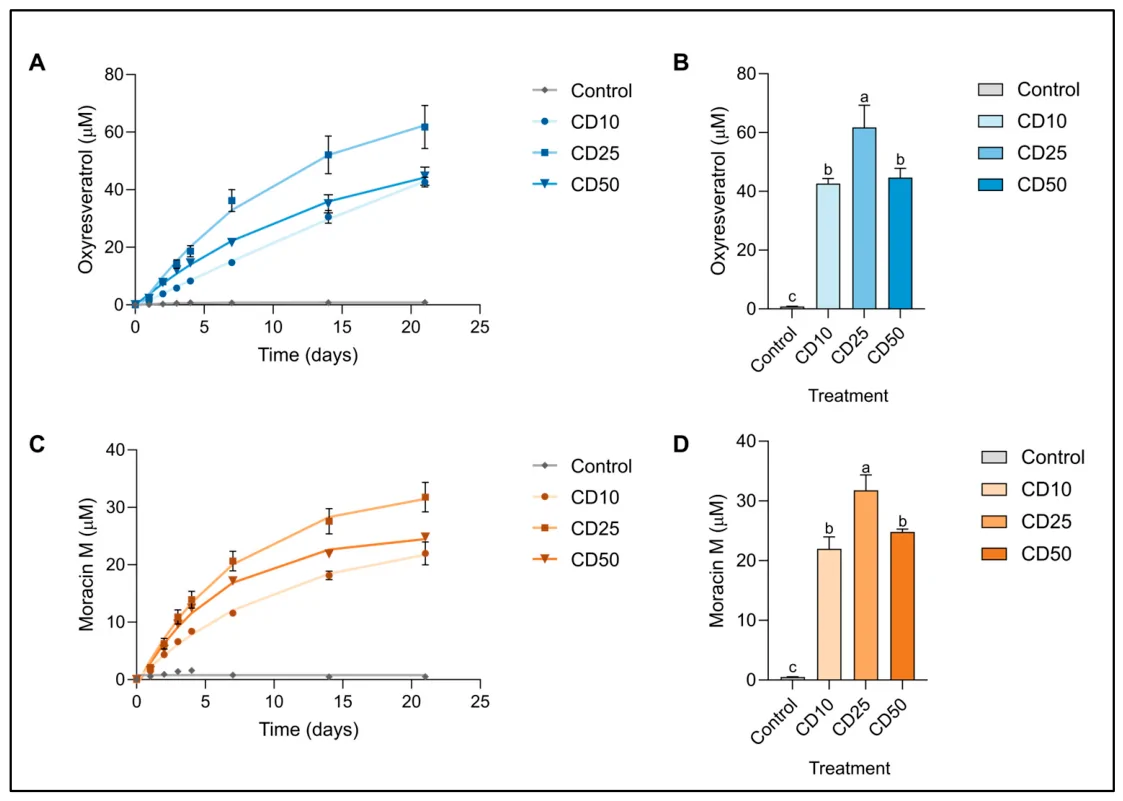
Time-course and final extracellular accumulation of oxyresveratrol and moracin M in *M. alba* adventitious root cultures elicited with HP-β-CD. (**A**) Accumulation kinetics of oxyresveratrol. (**B**) Final oxyresveratrol concentration after 21 days of elicitation. (**C**) Accumulation kinetics of moracin M. (**D**) Final moracin M concentration after 21 days of elicitation. Symbols and bars represent experimental data expressed as mean ± SEM (*n* = 4). Solid lines in panels (**A**,**C**) represent fits of the exponential accumulation model. Fitted parameters and standard errors, together with R^2^ values, are provided in Table S1. Different letters in panels (**B**,**D**) indicate statistically significant differences among treatments (one-way ANOVA followed by Tukey’s multiple comparisons test, *p* < 0.05).

Oxyresveratrol remained at very low levels in control cultures throughout the experiment. In contrast, all HP-β-CD treatments markedly increased extracellular oxyresveratrol accumulation over time (Figure 2A). The accumulation profiles of the elicited cultures were described by an exponential approach-to-asymptote model. The estimated apparent rate constants were 0.0126, 0.0886 and 0.0703 day^−1^ at 10, 25 and 50 mM, respectively. Parameter estimates and their standard errors are provided in Table S1. No clear plateau was observed for oxyresveratrol at 10 mM within 21 days, limiting interpretation of the estimated asymptote.

At the end of the experiment, oxyresveratrol concentrations were approximately 50-, 73- and 53-fold higher than the control in cultures treated with 10, 25 and 50 mM HP β-CD, respectively (Figure 2B). All elicited cultures accumulated significantly more oxyresveratrol than the control. Among the elicited treatments, the 25 mM HP-β-CD condition produced significantly higher oxyresveratrol than the 10 and 50 mM treatments, which did not differ significantly from each other.

#### 2.2.2. Moracin M Accumulation

Moracin M showed a distinct accumulation pattern in control cultures, reaching a maximum concentration of approximately 1.60 μM at day 4 and subsequently declining to 0.52 μM by day 21 (Figure 2C). The exponential accumulation model was not applicable to this non-monotonic profile. In contrast, extracellular moracin M concentrations increased progressively in all HP-β-CD-treated cultures, and their profiles were described by an exponential approach-to-asymptote model. The estimated apparent rate constants were 0.0930, 0.1351 and 0.1630 day^−1^ at 10, 25 and 50 mM HP-β-CD, respectively. Parameter estimates and their standard errors are provided in Table S1.

At the end of the experiment, moracin M concentrations were approximately 42-, 61- and 48-fold higher than the control in cultures treated with 10, 25 and 50 mM HP-β-CD, respectively (Figure 2D). All elicited cultures accumulated significantly more moracin M than the control. Among the elicited treatments, the 25 mM HP-β-CD condition produced significantly higher moracin M than the 10 and 50 mM treatments, which did not differ significantly from each other.

Representative HPLC chromatograms are shown in Figure S1. The elicited extracts displayed similar major peak patterns, with differences in peak area among treatments.

### 2.3. Total Phenolic Accumulation and Antioxidant Capacity of Elicited Adventitious Root Cultures

#### 2.3.1. Total Phenolic Content Assessed by the Folin–Ciocalteu Assay

**Figure 3:**
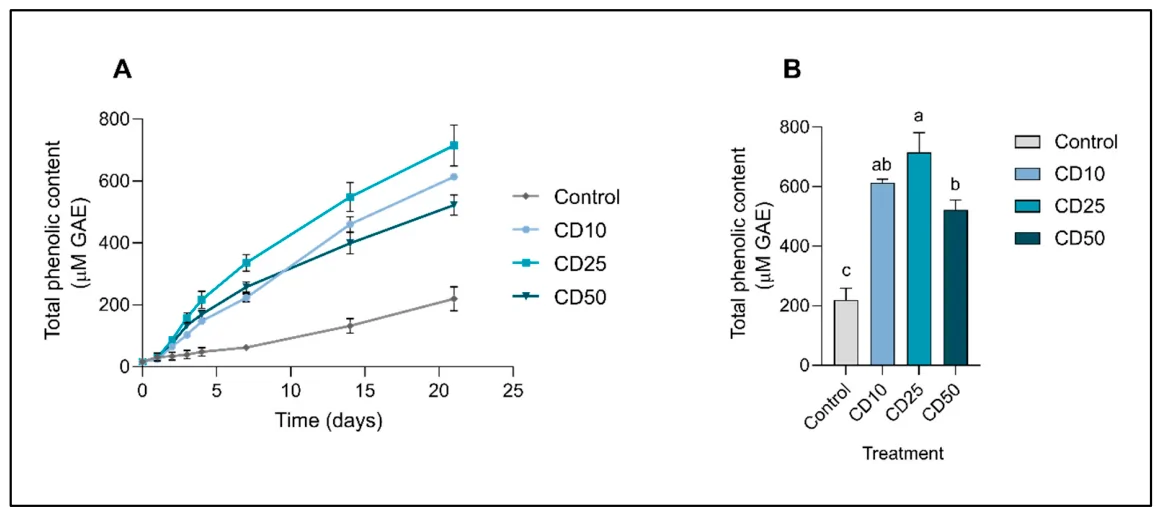
TPC in HP-β-CD-elicited *M. alba* adventitious root cultures. (**A**) Time-course of total phenolic content in culture supernatants, determined by the Folin–Ciocalteu assay and expressed as gallic acid equivalents (µM GAE) in the original culture supernatant. (**B**) TPC at day 21. Data represent mean values ± SEM (*n* = 4). Different letters indicate statistically significant differences among treatments (one-way ANOVA followed by Tukey’s multiple comparisons test, *p* < 0.05).

TPC was measured in culture supernatant extracts throughout the experiment using the Folin–Ciocalteu assay and expressed as gallic acid equivalents relative to the original culture supernatant volume. All treatments showed a progressive increase in TPC over time (Figure 3A). The highest TPC values were generally observed at 25 mM HP-β-CD. The relative ordering of the 10 and 50 mM treatments changed over time, with higher values at 10 mM at days 14 and 21. TPC continued to increase through day 21 in all treatments. By contrast, control cultures showed a slower initial increase, followed by a more marked rise during the final two weeks of culture.

At the end of the experiment, all elicited cultures showed significantly higher total phenolic content than control cultures (Figure 3B). The 25 mM treatment showed a significantly higher value than the 50 mM treatment, whereas the 10 mM treatment did not differ significantly from either of them.

#### 2.3.2. Antioxidant Capacity Determined by the ABTS Assay

Antioxidant capacity was determined in culture supernatant extracts collected at day 21 using the ABTS assay and expressed as Trolox equivalents relative to the original culture supernatant volume (Table 1). All elicited extracts showed significantly higher antioxidant capacity than the control. The highest value was observed for the 25 mM HP-β-CD treatment, followed by the 10 and 50 mM treatments. Antioxidant capacity was significantly higher at 25 mM than at 50 mM HP-β-CD, whereas no significant differences were detected between the 25 and 10 mM treatments or between the 10 and 50 mM treatments.

### 2.4. Effects on MCF-7 Cell Viability

**Figure 4:**
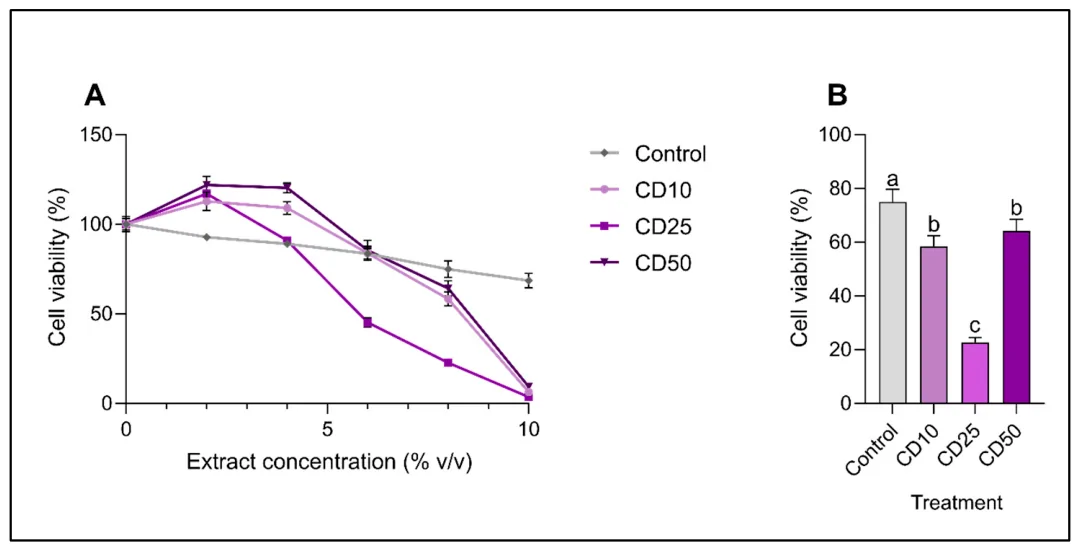
Effects of culture supernatant extracts on MCF-7 cell viability. (**A**) Dose–response curves showing the effect of culture supernatant extracts derived from non-elicited control cultures and HP-β-CD-elicited adventitious root cultures (CD10, CD25 and CD50). Cell viability is expressed as a percentage relative to vehicle-treated control cells and plotted as a function of extract concentration (% *v*/*v*). (**B**) Cell viability at 8% (*v*/*v*) extract concentration. Extract preparations contained pooled material from four biological replicate flasks per condition. Data represent mean values ± SEM of three independent experiments (*n* = 3), each with six technical replicate wells per condition. Different letters in panel (**B**) indicate statistically significant differences among treatments (one-way ANOVA followed by Tukey’s multiple comparisons test, *p* < 0.05).

The effect of culture supernatant extracts on MCF-7 cell viability was evaluated by MTT assay after 48 h of treatment over a concentration range of 0–10% (*v*/*v*) (Figure 4A).

Extracts obtained from non-elicited cultures produced a gradual decrease in cell viability as extract concentration increased. Extracts derived from HP-β-CD-elicited cultures showed an initial increase in MTT signal at low extract concentrations, followed by a decrease at higher concentrations. The decrease occurred at lower extract concentrations for CD25-derived extracts than for CD10- and CD50-derived extracts.

At 6% extract concentration, CD25-derived extracts reduced cell viability more strongly than control, CD10 and CD50 extracts. At 8% extract concentration, all elicited extracts reduced cell viability more than control extracts, with the CD25-derived extract reducing viability to 22.6% of the vehicle control after 48 h (Figure 4B). At 10% extract concentration, all elicited extracts produced an almost complete loss of cell viability, whereas control extracts produced a partial reduction. Osmolality controls did not produce detectable changes in MCF-7 cell viability under the experimental conditions used.

**Figure 5:**
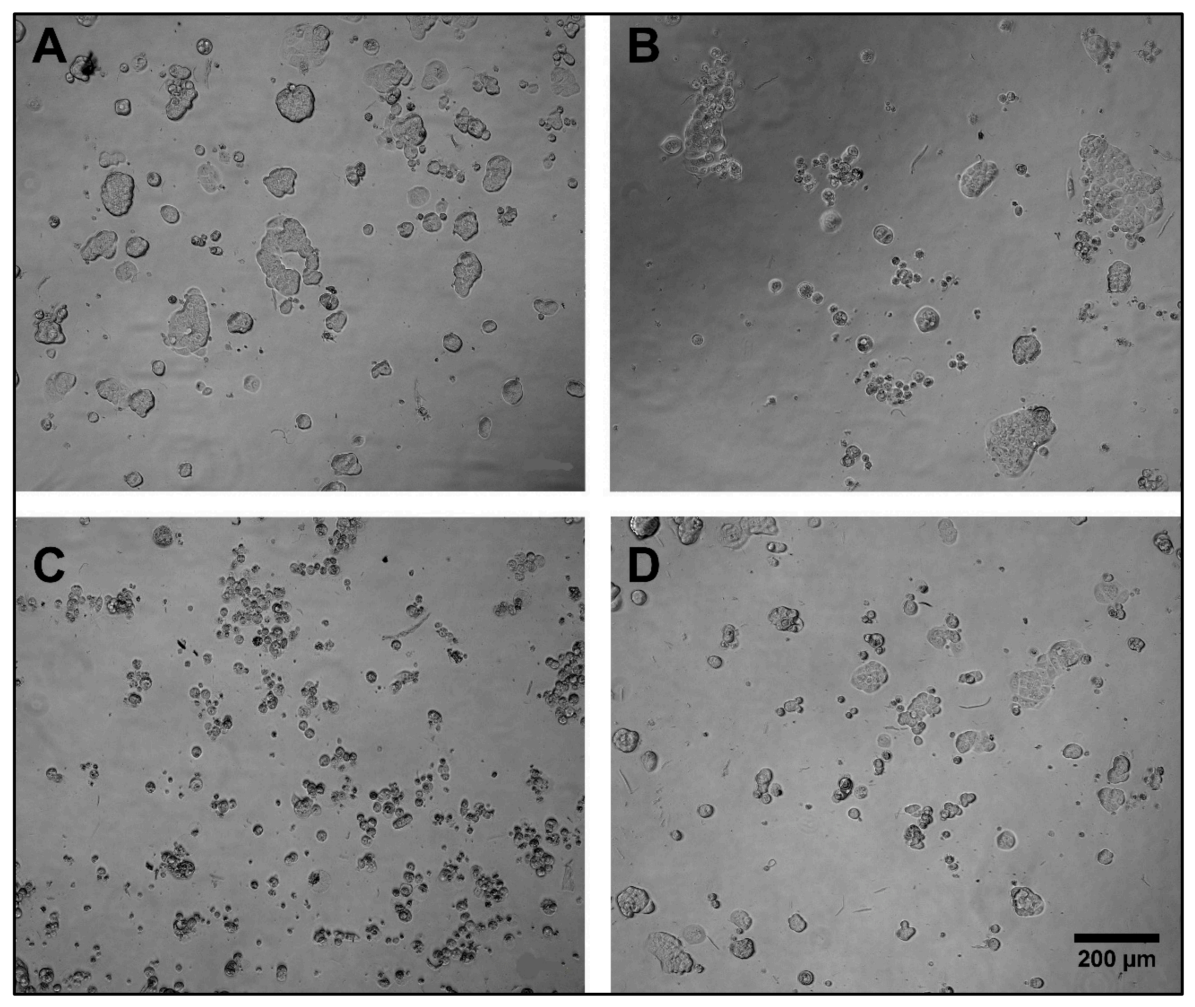
Phase-contrast microscopy of MCF-7 cells treated with culture supernatant extracts. Representative phase-contrast microscopy images of MCF-7 cells after 48 h of treatment with culture supernatant extracts at 8% (*v*/*v*). (**A**) Control extract from non-elicited root cultures, (**B**) CD10-derived extract, (**C**) CD25-derived extract and (**D**) CD50-derived extract.

Phase-contrast microscopy was performed after 48 h of treatment with 8% (*v*/*v*) extracts (Figure 5). Cells treated with non-elicited culture extracts retained visible cell aggregates and cell attachment, although rounded cells were also present. Cells treated with CD10- and CD50-derived extracts showed smaller cell aggregates and a higher proportion of rounded, weakly attached cells. Cells treated with CD25-derived extracts showed extensive loss of cell adhesion and very few organised cell aggregates.

## 3. Discussion

These results show that HP-β-CD elicitation altered both growth and extracellular phenolic accumulation in *M. alba* adventitious root cultures. Elicited roots accumulated less biomass but generated culture supernatants enriched in oxyresveratrol, moracin M and total phenolics, with increased antioxidant capacity and stronger effects on MCF-7 cell viability. This overall pattern links the elicitor-associated stress response in the plant culture with measurable downstream bioactivity in the resulting extracellular extracts.

The marked reduction in biomass observed after HP-β-CD treatment is consistent with a stress-associated response to elicitation. Elicitors are commonly used to mimic biotic or abiotic stress in plant in vitro cultures, thereby stimulating the biosynthesis and accumulation of secondary metabolites [16,17]. This response can involve a trade-off between growth-related processes and defence-associated specialised metabolism, which could contribute to the increased phenolic accumulation observed alongside reduced biomass. A similar inhibitory effect of CD treatment on biomass accumulation has also been reported in mulberry in vitro plants by Sabater-Jara et al. [11], supporting the possibility that CD-mediated elicitation imposes a metabolic cost on growth. However, neither DW_f_ nor the visual appearance of the cultures allows growth inhibition to be distinguished from loss of root viability. As root viability was not assessed directly, the relative contributions of cytostatic and cytotoxic effects remain unresolved. In the present study, no significant differences in root DW_f_ were detected among HP-β-CD treatments, whereas extracellular phenolic accumulation differed substantially. Thus, final biomass alone does not account for the differences in extracellular metabolite accumulation among elicited cultures.

CDs can favour extracellular phenolic accumulation through complementary biological and physicochemical effects. In plant culture systems, CD treatments have been associated with enhanced specialised metabolite production, while inclusion complex formation can retain hydrophobic metabolites in the aqueous medium and reduce their degradation [9,18]. Complexation lowers the freely available extracellular fraction and may favour further metabolite release and accumulation [9]. These mechanisms provide a plausible interpretation of the present results, but increased extracellular concentrations alone do not distinguish enhanced biosynthesis from altered release or stability. The molecular mechanism of HP-β-CD action in these adventitious roots was not directly investigated.

Against this background of growth inhibition, HP-β-CD markedly promoted the extracellular accumulation of both oxyresveratrol and moracin M. However, the two compounds showed distinct accumulation patterns. Moracin M displayed numerically higher apparent rate constants but reached lower final concentrations than oxyresveratrol, which accumulated more gradually and continued increasing throughout the experimental period. This behaviour is consistent with the proposed downstream position of moracin M in the stilbenoid pathway. Oxyresveratrol has been shown to undergo enzymatic conversion into moracin M in *M. alba* root extracts [13], and the faster but quantitatively more limited accumulation of moracin M may therefore reflect the rapid formation of a downstream product constrained by the conversion step or by subsequent metabolism. In contrast, the more sustained accumulation of oxyresveratrol may reflect continued flux into the upstream stilbenoid pool. Nevertheless, because only extracellular concentrations were measured, these patterns may also be influenced by compound-specific differences in release, CD-mediated solubilisation and extracellular stability [8], and they cannot establish pathway flux directly.

Among the tested conditions, 25 mM HP-β-CD produced the most favourable overall response. This treatment yielded the highest final concentrations of oxyresveratrol and moracin M, together with the highest total phenolic content and antioxidant capacity. At this concentration, oxyresveratrol reached 61.79 μM, corresponding to approximately 15.09 mg L^−1^, while moracin M reached 31.78 μM, approximately 7.70 mg L^−1^. These values compare favourably with previous *M. alba* in vitro production systems. In CD-elicited *M. alba* in vitro plants, extracellular oxyresveratrol levels of 5.62–7.74 mg L^−1^ were reported after 21 days of treatment [11], whereas multielicitor-treated hairy root cultures reached 8.67 mg L^−1^ oxyresveratrol and 10.33 mg L^−1^ moracin M in the culture medium [10]. Therefore, the present system achieved higher extracellular oxyresveratrol accumulation using HP-β-CD as a single elicitor, while producing substantial moracin M levels that approached those obtained under multielicitor conditions.

The differences observed among these studies may be partly attributable to the plant genotype, since the material used in the present work was selected following a previous screening of mulberry varieties for favourable phytochemical and productive traits [19]. Genotype-dependent variation can markedly influence both the basal production of specialised metabolites and the magnitude of the response to elicitation. In addition, the use of adventitious roots rather than whole in vitro plants may have contributed to the higher metabolite yields, as preliminary results from our experimental system indicated that roots accumulated more than tenfold higher levels of bioactive compounds than leaves under comparable conditions [20]. Differences in culture type, elicitor composition and concentration, treatment duration, biomass-to-medium ratio, sampling time and analytical methodology may also account for the quantitative discrepancies with the studies of Fang et al. [10] and Sabater-Jara et al. [11].

Furthermore, although 50 mM CD has been used in previous *M. alba* elicitation studies and in other plant culture systems [9,11,18], this concentration did not maximise extracellular metabolite accumulation in our adventitious root system. Instead, the response was non-linear, suggesting that an intermediate CD concentration provided sufficient stimulation of defence-associated metabolism, whereas the highest concentration may have imposed additional constraints on sustained biosynthesis, release or accumulation [17]. This finding also highlights the importance of performing a concentration-response screening rather than directly adopting a single dose previously reported in the literature, since the optimal elicitor concentration may vary according to the genotype, culture system and target metabolite. Thus, the experimental evaluation of several HP-β-CD concentrations was essential to identify 25 mM as the most effective condition for this specific adventitious root system.

The compound-specific behaviour was also reflected at the extract level. HP-β-CD increased both TPC and ABTS radical-scavenging capacity, indicating that the enhanced accumulation of the two quantified metabolites occurred within a wider enrichment of the extracellular phenolic fraction. Previous analyses of elicited *M. alba* root cultures have identified complex mixtures of stilbenes, benzofurans and related derivatives in the culture medium [10]. In this context, the Folin–Ciocalteu and ABTS assays provided complementary global readouts of the chemical enrichment and antioxidant functionality of the extracts [21]. ABTS antioxidant capacity was assessed only at day 21 to compare the final extracts obtained under each elicitation condition. Therefore, the agreement between TPC and ABTS observed at this endpoint cannot be extrapolated to their temporal profiles.

The effects of these chemically distinct extracts were subsequently evaluated in MCF-7 cells. Extracts from elicited cultures produced a non-monotonic response, characterised by an initial increase in MTT signal at low concentrations followed by a pronounced reduction at higher concentrations. This low-concentration increase was not observed with extracts from non-elicited cultures, which instead produced a gradual decline in cell viability. Since all treatments contained the same final DMSO concentration and the corresponding osmolality controls did not affect cell viability, these differences are unlikely to result from solvent- or osmolality-related effects. Because the extracts were tested at the same volumetric concentrations, the experimental design allowed direct comparison of the biological effects associated with the chemical profiles generated under each elicitation condition. The observed pattern is compatible with a hormetic-like response, as described for phenolic compounds and complex plant matrices, in which low exposure levels may stimulate adaptive cellular responses, whereas higher levels impair cellular function [22,23]. However, oxyresveratrol and related stilbenes exhibit phytoestrogenic activity, and MCF-7 cells are oestrogen receptor (ER)-positive; therefore, ER-mediated stimulation is a plausible alternative explanation for the increased MTT signal at low extract concentrations, although this mechanism was not investigated in the present study [24]. Because the MTT assay primarily reflects cellular metabolic activity rather than proliferation directly [25], the increase observed at low extract concentrations should be interpreted as an enhanced viability-associated metabolic signal rather than unequivocal stimulation of cell growth.

The reduction in MCF-7 cell viability observed here is consistent with previous reports describing cytotoxic or pro-apoptotic effects of conventional *M. alba* extracts in breast cancer models. Methanolic leaf extracts of *M. alba* have been shown to inhibit MCF-7 cell viability and induce apoptosis-associated responses, while root extracts have been reported to reduce the viability of both MCF-7 and multidrug-resistant MCF-7/Dox cells [26,27]. However, these studies used extracts obtained from plant tissues rather than extracellular fractions generated through controlled elicitation. Conversely, elicited plant culture systems from other species have shown that enhanced phenolic accumulation can be accompanied by stronger cytotoxic effects in cancer cell models, including MCF-7 cells [28,29]. In this context, the present work provides a distinct production-to-function approach by linking CD-mediated phenolic enrichment in *M. alba* adventitious root culture supernatants with measurable effects in a breast tumour cell model.

The non-monotonic curves also limit the usefulness of conventional IC_50_ values, since a single midpoint would not adequately represent the initial increase in MTT signal followed by the subsequent loss of viability. Comparison at 8% (*v*/*v*) was therefore more informative for distinguishing the biological effects of the extracts before the near-complete loss of viability observed at 10%, where a floor effect reduced discrimination among treatments. At 8%, all elicited extracts reduced MCF-7 cell viability more strongly than the non-elicited extract, with the CD25-derived extract producing the greatest effect. This response was consistent with the higher phenolic content measured in the analytical extracts from the same elicitation condition. However, although both preparations involved ethyl acetate extraction, analytical extracts were reconstituted in methanol, whereas cell-assay extracts were reconstituted in DMSO and contained pooled material from four biological replicate flasks. This agreement therefore represents an association across elicitation conditions rather than a direct quantitative relationship between the phenolic content of individual extracts and their effects on cell viability. The effect should not, however, be attributed to a single constituent. Oxyresveratrol has been reported to affect MCF-7 cells through mechanisms involving apoptosis and cell-cycle regulation [15], whereas direct evidence for moracin M in breast cancer models remains limited. Related moracin derivatives have nevertheless shown cytotoxic activity in breast cancer cells [30], supporting the possible contribution of this compound class. More broadly, the response is likely to reflect the combined activity of the elicitation-enriched extracellular metabolite profile rather than either quantified compound alone.

The phase-contrast images provided a phenotypic counterpart to the MTT results. At 8% (*v*/*v*), cells treated with elicited extracts showed reduced attachment, increased rounding and loss of organised aggregates, with the most extensive alterations observed for the CD25-derived extract. The agreement between morphological and metabolic responses supports the conclusion that elicitation-dependent differences in extract composition produced genuine changes in cellular status. In adherent cells, disruption of cell–substrate interactions can contribute to detachment-associated loss of viability [31]. Nevertheless, morphology alone cannot distinguish among growth arrest, detachment-associated loss of viability, apoptosis or other forms of cell death, and mechanistic assays would be required to identify the processes involved.

Taken together, these findings support optimising HP-β-CD elicitation by considering both extracellular metabolite production and the biological properties of the resulting extracts. Within the tested range, 25 mM HP-β-CD combined the highest extracellular phenolic output and antioxidant capacity with the strongest reduction in MCF-7 cell viability at the selected extract concentration. Despite the associated reduction in root growth, this single-elicitor strategy enabled the production of phenolic-rich culture supernatants from *M. alba* adventitious roots, highlighting the extracellular fraction as a relevant endpoint for culture optimisation and subsequent functional evaluation.

## 4. Materials and Methods

### 4.1. Plant Material and Establishment of Morus alba Adventitious Root Cultures

Plant material was collected from 20-year-old mulberry (*Morus alba* L.) trees growing at the experimental field station of the Instituto Murciano de Investigación y Desarrollo Agrario y Alimentario (IMIDA, Murcia, Spain). Nodal segments of the scion cultivar ‘Filipina’, a variety previously characterised for its phenolic profile and bioactive potential [19], were collected and transferred to the tissue culture laboratory at the beginning of spring.

The nodal segments were sterilised in a solution of sodium dichloroisocyanurate (NaDCC, 2.7 g L^−1^; Sigma-Aldrich, Steinheim, Germany) under agitation for 90 min. Sterilised segments were aseptically trimmed and individually introduced into test tubes containing culture media for shoot culture establishment. The medium was composed of Murashige and Skoog (MS) salts [32], 3% (*w*/*v*) sucrose and 0.7% (*w*/*v*) of Plant Agar (Duchefa Biochemie, Haarlem, NL, USA). The pH was adjusted to 5.7 with 0.1 N KOH prior to autoclaving at 122 °C (1.1 kg cm^−2^) for 16 min. The proliferating shoots were cultured in climatic chambers at 25 ± 1 °C and with a 16 h light period (45 μmol m^−2^ s^−1^ white LED light [380–780 nm]).

Cultures were maintained for 4 weeks and subcultured into fresh individual tubes to ensure the establishment of pathogen-free in vitro plantlets. Axillary buds from healthy cultures were subsequently transferred to culture vessels at a density of 10 buds per vessel for further multiplication.

The multiplication and root-inducing medium consisted of MS medium supplemented with 0.5 mg L^−1^ 1-naphthaleneacetic acid (NAA), 0.5 mg L^−1^ 6-benzylaminopurine, 3% (*w*/*v*) sucrose, 0.6% (*w*/*v*) Plant Agar (Duchefa Biochemie, Haarlem, the Netherlands), and 2.5 g L^−1^ activated charcoal. The pH was adjusted to 5.7 with 0.1 N KOH prior to autoclaving at 122 °C (1.1 kg cm^−2^) for 16 min. The proliferating shoots were cultured in climatic chambers at 25 ± 1 °C and with a 16 h light period (45 μmol m^−2^ s^−1^ white LED light [380–780 nm]).

### 4.2. Cyclodextrin-Mediated Elicitation and Sampling of Adventitious Root Cultures

Adventitious root cultures derived from 4-week in vitro-grown *M. alba* plantlets were used for elicitation experiments. Actively growing root apices (1 cm in length) were excised and used as explants. An initial fresh weight of 0.25 g of root apices was transferred into 250 mL Erlenmeyer flasks containing 150 mL of liquid Woody Plant Medium (WPM) [33] supplemented with 3% (*w*/*v*) sucrose and 1 mg L^−1^ NAA [20]. The pH was adjusted to 5.7 with 0.1 N KOH prior to autoclaving at 122 °C (1.1 kg cm^−2^) for 16 min. All elicitation experiments were performed using four independent biological replicates.

Elicitation was carried out by supplementing the culture medium with HP-β-CD at final concentrations of 0 (control), 10, 25, and 50 mM [11]. The CD used was CAVASOL^®^ W7 (Wacker Chemie AG, Munich, Germany), with an average molecular weight of 1510 g mol^−1^, a purity of 96%, and a degree of substitution of 0.9.

Cultures were maintained in constant darkness at 25 ± 1 °C under continuous orbital agitation at 90 rpm throughout the experimental period. Adventitious root cultures were grown for a total of 21 days. During the early phase of the experiment, aliquots (1 mL) of the culture medium were collected at 0, 24, 48, 72, and 96 h to monitor the initial accumulation of extracellular metabolites. Additional samples were collected after 7, 14 and 21 days of culture to evaluate long-term metabolite production. Culture supernatants were stored at −20 °C until further analysis.

### 4.3. Biomass Determination of Adventitious Root Cultures

Initial fresh weight (FW_0_) was recorded at the start of the elicitation experiments. At the end of the elicitation period, adventitious roots were recovered by decantation using a mesh strainer and thoroughly rinsed with distilled water. Roots were gently blotted dry with filter paper and immediately weighed to determine final fresh weight using a precision balance (±0.1 mg). Samples were subsequently frozen and lyophilised, and dry weight (DW_f_) was recorded after freeze-drying. All measurements were performed using four independent biological replicates.

### 4.4. HPLC Analysis and Quantification of Phenolic Compounds

Extracellular metabolites were extracted from culture supernatants collected at the different sampling times following a previously described procedure [34] with slight modifications. Aliquots (1 mL) of culture medium were thawed and subjected to liquid-liquid extraction by the addition of 1 mL of ethyl acetate (Fisher Scientific, Madrid, Spain) directly in 2 mL microtubes. Samples were vortexed for 1 min and subsequently centrifuged at 13,600× *g* for 5 min at room temperature using a Spectrafuge 24D centrifuge (Labnet International, Edison, NJ, USA) to ensure efficient phase separation and removal of CDs.

The organic phase was carefully transferred to 1.5 mL microtubes and evaporated to dryness under vacuum using an Eppendorf Concentrator 5301 (Eppendorf, Hamburg, Germany). The resulting residue was reconstituted in 200 µL of methanol, yielding a fivefold concentration relative to the original culture medium. Reconstituted extracts were directly used for HPLC analysis, as well as for total phenolic content and antioxidant activity assays. For the latter assays, appropriate dilutions were performed to avoid signal saturation.

Chromatographic analyses were performed using an Agilent 1200 Series HPLC system (Agilent Technologies, Santa Clara, CA, USA) equipped with a quaternary pump and a diode array detector (DAD). Separation was achieved on a Kromasil C18 column (150 × 4.6 mm, 5 µm particle size) fitted with a Supelco C18 guard column (1 mm, 5 µm; Análisis Vínicos, Tomelloso, Spain). The mobile phase consisted of water (solvent A) and acetonitrile (solvent B), both containing 0.1% (*v*/*v*) glacial acetic acid, delivered at a flow rate of 1 mL min^−1^. All HPLC-grade solvents were obtained from Fisher Scientific (Spain).

The gradient elution programme was as follows: 0–15 min, 24% B; 16–24 min, 99% B; 25–30 min, 24% B. The injection volume was 10 µL. The autosampler tray was maintained at 4 °C, while the column oven was set at 25 °C.

Oxyresveratrol and moracin M were identified by comparison of retention times and UV-visible spectra with authentic standards. Extracts were purified using a Shimadzu Nexera Prep system (LC-20AP pump; SPD-M40 PDA detector; Shimadzu Corporation, Kyoto, Japan) with a Kinetex C18 column (250 × 21.2 mm, 5 μm; Phenomenex, Torrance, CA, USA). Water and acetonitrile, both containing 0.1% (*v*/*v*) acetic acid, were used with the analytical HPLC gradient described above, at 25 mL min^−1^ with a 5 mL injection volume. Fractions were collected automatically using collection windows established from a preliminary injection. Compound identity was confirmed by HPLC-ESI-MS in negative ion mode using an Agilent iFunnel 6550 QTOF system.

Quantification was carried out using external calibration curves prepared for each compound in methanol over a concentration range of 0.5–250 µM. Calibration standards were injected together with the samples, and periodic quality-control injections were included to monitor analytical reproducibility. No detector saturation was observed under the selected analytical conditions, and calibration curves showed excellent linearity across the tested concentration range. Oxyresveratrol standard (>98% purity) was obtained from TCI (Zwijndrecht, Belgium), and moracin M standard (≥99.8% purity) from TargetMol (Boston, MA, USA).

### 4.5. Kinetic Modelling of Oxyresveratrol and Moracin M Accumulation

Time-course data of extracellular oxyresveratrol and moracin M accumulation were fitted independently for each compound and elicitation treatment using the mean concentration at each sampling time. Accumulation profiles were described using the exponential model:

*C*
(*t*) = *A* − *B* · *e*^−*k*·*t*^, (1)

where *C*(*t*) represents the concentration of the compound at time *t*, *A* is the estimated asymptotic concentration, *B* is the amplitude parameter, and *k* is the apparent rate constant.

Parameters were estimated by unweighted nonlinear least-squares regression using GraphPad Prism version 9.0.0. Parameter standard errors were calculated using a leave-one-flask-out jackknife procedure based on four biological replicate time courses. Each flask was omitted in turn, and the model was refitted to the mean profile of the remaining flasks, retaining the complete time course of each included flask.

Goodness of fit was assessed using *R*^2^, calculated as 1-SSE/SST, where SSE is the sum of squared differences between the mean experimental concentrations and model predictions, and SST is the sum of squared deviations of the mean experimental concentrations from their overall mean. Parameter estimates, jackknife standard errors and *R*^2^ values are reported in Table S1.

### 4.6. Determination of Total Phenolic Content and Antioxidant Capacity

Total phenolic content (TPC) and antioxidant capacity were determined using the same organic extracts prepared for HPLC-DAD analysis. After reconstitution of the dried extracts in methanol, aliquots were directly used for chromatographic analysis, while the remaining volume was employed for Folin–Ciocalteu and ABTS assays [21].

#### 4.6.1. Total Phenolic Content (Folin–Ciocalteu Assay)

TPC was determined using the Folin–Ciocalteu method adapted to a 96-well microplate format, essentially as previously described in the literature [35]. Briefly, 7.5 µL of sample extract were mixed with 7.5 µL of acidified water (prepared by adding 34 µL of 35% hydrochloric acid per mL of water), 170 µL of HPLC-grade water, and 12 µL of Folin-Ciocalteu reagent (Merck, Rahway, NJ, USA). The reaction was initiated by the addition of 30 µL of sodium carbonate solution (200 g L^−1^) and incubated for 1 h in the dark at room temperature. Subsequently, 73 µL of HPLC-grade water were added, and absorbance was measured at 765 nm using a Synergy HT microplate reader (BioTek Instruments, Winooski, VT, USA).

Gallic acid was used as the calibration standard. Calibration curves were prepared to yield final concentrations in the reaction well ranging from 10 to 100 µM, corresponding to initial gallic acid solutions of 0.4–4 mM prior to dilution in the microplate. TPC was expressed as gallic acid equivalents (GAE). Samples were analysed at appropriate dilution factors to ensure measurements within the linear range of the assay. Control samples and elicited cultures collected up to 7 days were analysed at fivefold the original supernatant concentration. Extracts from elicited cultures collected at days 14 and 21 were diluted from fivefold to twofold the original supernatant concentration before analysis.

#### 4.6.2. Antioxidant Capacity (ABTS Assay)

Antioxidant capacity was determined using the ABTS radical cation decolourisation assay [36]. The ABTS^•+^ radical was generated enzymatically by dissolving ABTS (20 mM) in water and mixing it with 0.2 M acetate buffer (pH 5.0) and ultrapure water. Radical formation was initiated by the addition of hydrogen peroxide and horseradish peroxidase type VI (HRP-VI), followed by gentle mixing and incubation for 1 h in the dark at room temperature.

The reaction conditions were adjusted to obtain an ABTS^•+^ working solution with suitable absorbance. Radical formation was verified spectrophotometrically by measuring the absorbance of a 1:10 (*v*/*v*) dilution, which yielded a value of 0.30 at 414 nm.

For the assay, 200 µL of the ABTS^•+^ solution were combined with 80 µL of 0.2 M phosphate buffer (pH 7.0) and 20 µL of sample solution in 96-well microplates. After incubation for 5 min in the dark at room temperature, absorbance was measured at 414 nm using the same Synergy HT microplate reader described above.

Samples were diluted in methanol prior to analysis to ensure measurements within the linear range of the Trolox calibration curve (0–25 µM in the reaction well). Dilutions of 0.05× and 0.1× were analysed, and proportional responses between dilutions were verified. Each dilution was measured in triplicate using extracts from four independent biological replicates.

### 4.7. Cell Culture and Cell Viability Assessment in MCF-7 Cells

The human breast cancer cell line MCF-7 was obtained from the American Type Culture Collection (ATCC) and maintained under standard culture conditions. Cells were grown in Eagle’s Minimum Essential Medium (EMEM) supplemented with 10% foetal bovine serum (FBS), insulin (0.01 mg mL^−1^), penicillin (100 U mL^−1^) and streptomycin (100 μg mL^−1^). Cultures were maintained at 37 °C in a humidified atmosphere containing 5% CO_2_.

Cells were routinely subcultured under exponential growth conditions using standard procedures [37].

#### 4.7.1. Preparation of Extracts for Cell-Based Assays

For cell-based assays, culture supernatants obtained at the end of the elicitation experiments were collected in 50 mL centrifuge tubes and stored frozen until use. For each elicitation condition, the cell-assay preparations contained pooled material from four biological replicate flasks.

Aliquots of these supernatants were subjected to liquid–liquid extraction with ethyl acetate. The organic phase was recovered and evaporated to dryness under vacuum, and the resulting residue was reconstituted in dimethyl sulfoxide (DMSO) to obtain 20-fold concentrated stock solutions relative to the original supernatant volume.

These stock solutions (100% DMSO) were subsequently diluted to 5-fold concentrates in cell culture medium (25% DMSO), and further diluted in complete EMEM to obtain the desired final concentrations for cell treatments.

#### 4.7.2. MTT Cell Viability Assay

For cell viability assays, MCF-7 cells were seeded in 96-well plates at a density of 10,000 cells cm^−2^ and allowed to attach and reach exponential growth for 48 h prior to treatment. Peripheral wells were filled with sterile water to minimise evaporation effects [37].

Cells were treated with extracts prepared as described in Section 4.7.1 from non-elicited control cultures and cultures elicited with 10, 25 or 50 mM HP-β-CD. Extract doses were expressed as original culture supernatant volume equivalents, corresponding to 0, 2, 4, 6, 8 and 10% (*v*/*v*) in the final treatment medium. The final concentration of DMSO was adjusted to 0.50% (*v*/*v*) in all treatments, including vehicle controls, by the addition of appropriate volumes of DMSO.

The osmolality of the treatment solutions was measured using a Vapro 5520 vapour pressure osmometer (Wescor, Logan, UT, USA). Osmolality controls with sucrose covering the values measured in the extract treatments were included to confirm that differences in osmolality did not affect MCF-7 cell viability.

After 48 h of exposure to the treatments, culture media were completely replaced with fresh medium containing MTT (1 mg mL^−1^), followed by incubation for 4 h at 37 °C in the dark. The MTT-containing medium was then removed, and the resulting formazan crystals were solubilised in DMSO.

Absorbance was measured at 560 nm, with background correction at 690 nm, using a FLUOstar Omega microplate reader (BMG Labtech, Ortenberg, Germany). Cell viability was expressed as a percentage relative to vehicle-treated control cells. Each condition was analysed in technical sextuplicate within each plate, and experiments were independently repeated three times.

#### 4.7.3. Phase-Contrast Microscopy

Morphological changes in MCF-7 cells following treatment with culture supernatant extracts were monitored by phase-contrast microscopy using an inverted ZEISS Axio Observer 7 microscope (Carl Zeiss Microscopy GmbH, Jena, Germany). Representative images were acquired after 48 h of exposure to selected extract concentrations [38].

All images were captured under identical optical conditions and magnification.

### 4.8. Statistical Analysis

Statistical analyses were performed using GraphPad Prism 9.0.0 (GraphPad Software, San Diego, CA, USA). Data are presented as mean ± standard error of the mean (SEM). Data normality was evaluated using the Shapiro–Wilk test. Following assessment of normality, differences among elicitation treatments in final root dry weight, final extracellular oxyresveratrol and moracin M concentrations, total phenolic content and ABTS antioxidant capacity were analysed using one-way analysis of variance (ANOVA) followed by Tukey’s multiple comparisons test. The same analysis was used to compare MCF-7 cell viability among extract treatments at 8% (*v*/*v*). For cell viability assays, the six technical replicate wells were averaged within each independent experiment, and the resulting three independent means were used for statistical analysis and calculation of the SEM (*n* = 3). Statistical significance was set at *p* < 0.05.

## 5. Conclusions

HP-β-CD elicitation reduced final root biomass and increased extracellular oxyresveratrol and moracin M concentrations, total phenolic content and antioxidant capacity in *M. alba* adventitious root cultures. Among the conditions tested, 25 mM HP-β-CD produced the highest final concentrations of both target metabolites and generated the extract with the strongest effect on MCF-7 breast cancer cell viability at 8% (*v*/*v*).

By combining metabolite analysis, global extract characterisation and cell-based evaluation, this work identifies the culture supernatant as a relevant functional output of the elicitation process. The use of HP-β-CD as a single elicitor provides a simple and defined strategy to obtain phenolic-rich extracts, supporting the optimisation of elicitation conditions not only by metabolite yield but also by the biological properties of the resulting extracts.

## Figures and Tables

**Table 1:** Antioxidant capacity of culture supernatant extracts at the end of the elicitation period, assessed by ABTS radical scavenging assay.

Treatment	Antioxidant Capacity (μM Trolox Equivalents)
Control	59.26 ± 3.52 ^c^
CD10	162.24 ± 3.07 ^ab^
CD25	183.44 ± 6.03 ^a^
CD50	133.85 ± 5.53 ^b^

Values are expressed as μM Trolox equivalents in the original culture supernatant and presented as mean ± SEM (*n* = 4). Different superscript letters indicate statistically significant differences among treatments (one-way ANOVA followed by Tukey’s multiple comparisons test, *p* < 0.05).

## Data Availability

The raw data supporting the conclusions of this article will be made available by the authors on request.

## Supplementary Materials

The following supporting information can be downloaded at: https://www.mdpi.com/article/10.3390/plants15213255/s1, Figure S1: Representative HPLC chromatograms recorded at 310 nm of culture supernatant extracts collected after 21 days of elicitation from M. alba adventitious root cultures treated with (A) 0, (B) 10, (C) 25 and (D) 50 mM HP-β-CD. Oxyresveratrol and moracin M peaks are indicated. All main panels share the same axis scales. The inset in panel A shows the control chromatogram with a tenfold expanded vertical scale to improve the visibility of low-intensity peaks; Table S1: Parameters of the exponential model fitted to extracellular oxyresveratrol and moracin M accumulation in HP-β-CD-elicited *Morus alba* adventitious root cultures.
