# Supplementary material for: Bioactive Extracts from Cyclodextrin-Elicited Morus alba Roots Reduce MCF-7 Breast Cancer Cell Viability
Source: Plants (Basel). 2026 Oct 22;15(21):3255. doi: 10.3390/plants15213255 (PMC13766112; doi:10.3390/plants15213255)
Supplement: Supplementary file 1 [file plants-15-03255-s001.zip › plants-4617560-supplementary.pdf]

# Supplementary Materials

**Table S1.** Parameters of the exponential model fitted to extracellular oxyresveratrol and moracin M accumulation in HP-β-CD-elicited *Morus alba* adventitious root cultures.

Mean concentration profiles were fitted using $C(t) = A - B \cdot e^{-k \cdot t}$, where *C(t)* is the extracellular concentration (μM) at time t (days), *A* is the fitted asymptotic concentration (μM), *B* is the amplitude parameter (μM), and *k* is the apparent rate constant ($day^{-1}$). Parameter estimates are reported ± standard errors, calculated by leave-one-flask-out jackknife resampling of four biological replicates (n=4), retaining each flask's complete time course. Each flask was omitted in turn, and the model was refitted to the mean profile of the remaining three flasks. $R^2$ was calculated by comparing model predictions with the mean experimental concentrations at each sampling time.

| **Compound** | **HP-β-CD (mM)** | **A ± SE (μM)** | **B ± SE (μM)** | **k ± SE ($day^{-1}$)** | **$R^2$** |
|---|---|---|---|---|---|
| Oxyresveratrol | 10 | 187.23 ± 25.04* | 188.03 ± 24.86 | 0.0126 ± 0.0024 | 0.9988 |
| Oxyresveratrol | 25 | 74.33 ± 10.30 | 77.09 ± 10.72 | 0.0886 ± 0.0035 | 0.9928 |
| Oxyresveratrol | 50 | 57.34 ± 5.72 | 57.47 ± 5.94 | 0.0703 ± 0.0083 | 0.9967 |
| Moracin M | 10 | 25.36 ± 3.58 | 25.42 ± 3.41 | 0.0930 ± 0.0166 | 0.9966 |
| Moracin M | 25 | 33.52 ± 2.43 | 34.55 ± 2.37 | 0.1351 ± 0.0069 | 0.9951 |
| Moracin M | 50 | 25.33 ± 0.29 | 26.39 ± 0.27 | 0.1630 ± 0.0036 | 0.9904 |

* The asymptote for oxyresveratrol at 10 mM HP-β-CD represents an extrapolation beyond the observed sampling period and should be interpreted cautiously.

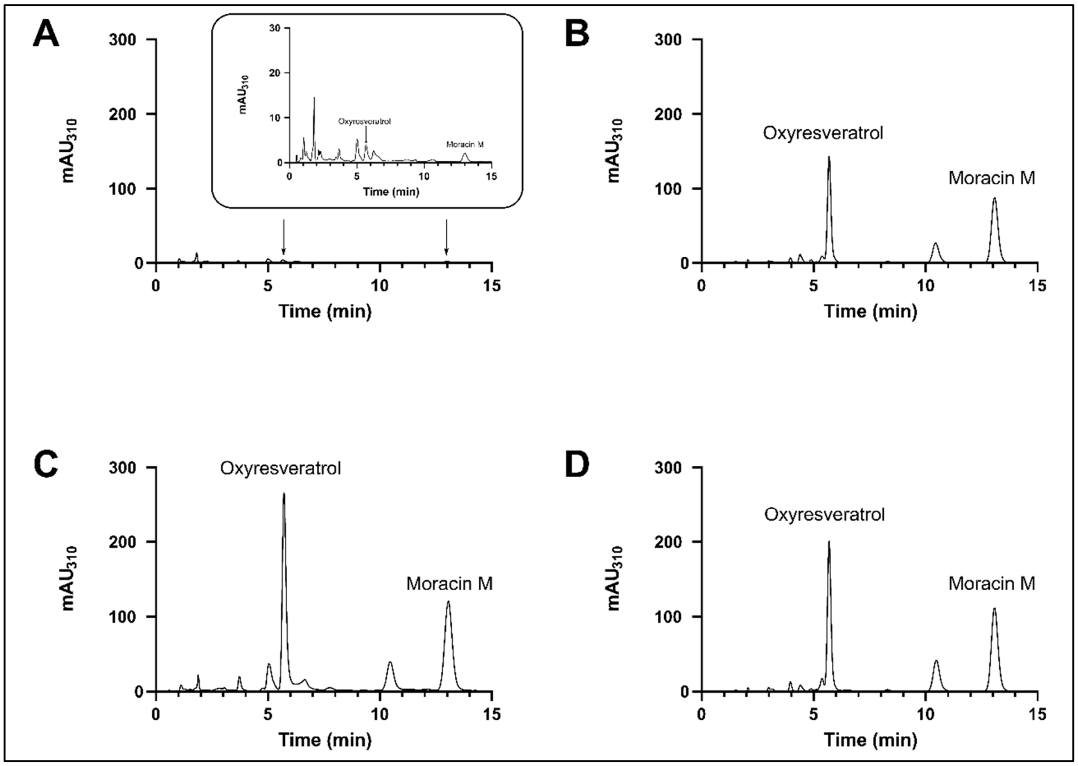


**Figure S1.** Representative HPLC chromatograms recorded at 310 nm of culture supernatant extracts collected after 21 days of elicitation from *M. alba* adventitious root cultures treated with (A) 0, (B) 10, (C) 25 and (D) 50 mM HP-β-CD. Oxyresveratrol and moracin M peaks are indicated. All main panels share the same axis scales. The inset in panel A shows the control chromatogram with a tenfold expanded vertical scale to improve the visibility of low-intensity peaks.
